# High cell density media for *Escherichia coli *are generally designed for aerobic cultivations – consequences for large-scale bioprocesses and shake flask cultures

**DOI:** 10.1186/1475-2859-7-26

**Published:** 2008-08-07

**Authors:** Jaakko Soini, Kaisa Ukkonen, Peter Neubauer

**Affiliations:** 1 Bioprocess Engineering Laboratory, Dept. of Process and Environmental Engineering, University of Oulu, P. O. Box 4300, FIN-90014 Oulu, Finland

## Abstract

**Background:**

For the cultivation of *Escherichia coli *in bioreactors trace element solutions are generally designed for optimal growth under aerobic conditions. They do normally not contain selenium and nickel. Molybdenum is only contained in few of them. These elements are part of the formate hydrogen lyase (FHL) complex which is induced under anaerobic conditions. As it is generally known that oxygen limitation appears in shake flask cultures and locally in large-scale bioreactors, function of the FHL complex may influence the process behaviour. Formate has been described to accumulate in large-scale cultures and may have toxic effects on *E. coli*.

Although the anaerobic metabolism of *E. coli *is well studied, reference data which estimate the impact of the FHL complex on bioprocesses of *E. coli *with oxygen limitation have so far not been published, but are important for a better process understanding.

**Results:**

Two sets of fed-batch cultures with conditions triggering oxygen limitation and formate accumulation were performed. Permanent oxygen limitation which is typical for shake flask cultures was caused in a bioreactor by reduction of the agitation rate. Transient oxygen limitation, which has been described to eventually occur in the feed-zone of large-scale bioreactors, was mimicked in a two-compartment scale-down bioreactor consisting of a stirred tank reactor and a plug flow reactor (PFR) with continuous glucose feeding into the PFR.

In both models formate accumulated up to about 20 mM in the culture medium without addition of selenium, molybdenum and nickel. By addition of these trace elements the formate accumulation decreased below the level observed in well-mixed laboratory-scale cultures. Interestingly, addition of the extra trace elements caused accumulation of large amounts of lactate and reduced biomass yield in the simulator with permanent oxygen limitation, but not in the scale-down two-compartment bioreactor.

**Conclusion:**

The accumulation of formate in oxygen limited cultivations of *E. coli *can be fully prevented by addition of the trace elements selenium, nickel and molybdenum, necessary for the function of FHL complex. For large-scale cultivations, if glucose gradients are likely, the results from the two-compartment scale-down bioreactor indicate that the addition of the extra trace elements is beneficial. No negative effects on the biomass yield or on any other bioprocess parameters could be observed in cultures with the extra trace elements if the cells were repeatedly exposed to transient oxygen limitation.

## Background

*Escherichia coli *is widely cultivated under aerobic conditions in laboratory and industrial processes. The standard procedure for growing *E. coli *cells to high cell densities is the fed-batch technique, where the carbon substrate, e.g. glucose, controls the growth as a limiting factor. To minimize the volume change in the bioreactor, high concentrated glucose solutions are often used. In large-scale cultivation processes mixing is often insufficient for equal distribution of the substrate resulting in gradients in essential variables such as substrate concentration, dissolved oxygen tension and pH [1].

Concentrations of glucose far above the saturation constant of the Monod model are connected to high metabolic and respiratory activities. Consequently, due to the low solubility of oxygen in aqueous solutions, the dissolved oxygen tension (DOT) drops to zero already at relatively low cell densities depending on the oxygen transfer rate of the cultivation system which is typical in shake flask cultures [2]. Also in large-scale glucose limited fed-batch processes with limited mixing high glucose concentrations in the feeding zone have been proposed [1]. At typical cell densities, ranging in such reactors from 10 to 100 g L^-1 ^of cell dry weight, the high local volumetric rates for consumption of glucose and oxygen easily cause oxygen depletion [3].

When exposed to oxygen limitation or anaerobic conditions *E. coli *shifts to anaerobic respiration, if the corresponding inorganic electron acceptors are available, or to fermentative metabolism. As a result of the fermentative metabolism oxidised fermentation products, such as formate, acetate, lactate, ethanol, and succinate are released to the cultivation medium and consequently the pH of the medium may decrease. The transition to oxygen limitation is rather sharp, as the oxygen uptake follows Michaelis Menten kinetics and the K_M _value for oxygen is very small, between 10^-7 ^and 10^-8 ^M for *E. coli *[4]. This correlates to less than 0.2% of oxygen saturation, which is so small that an exact analysis of the critical DOT level is not possible with standard DOT electrodes.

Indication of anaerobic metabolism in the glucose feeding zone of large-scale bioreactors came originally from studies in a scale-down two-compartment bioreactor system, where the synthesis of the above mentioned side metabolites was observed in the plug flow reactor compartment simulating the feeding zone [3,5]. Interestingly, despite the local oxygen limitation in the feeding zone, these anaerobic products are re-assimilated in the glucose limited, oxygen sufficient parts of the bioreactor, which normally make up more than 90% of the reactor volume [1,3]. The re-assimilation rates for acetate and lactate appear to be higher than for formate. Consequently, formate can be found as a side product in large-scale processes and is a clear indicator of anaerobic metabolism [1,6,7].

Additionally formate has been also found in small-scale processes where the DOT was kept above 30% and that therefore should be clearly aerobic. Castan et al. [8] showed that formate accumulation in these cultures was due to cell lysis. The authors verified by comparing their results to cultivations where a DNA binding polymer was added, that released DNA bound to cells was the reason of formate production. The DNA formed an extra diffusion barrier around the cells leading to decrease in oxygen transfer.

Physiologically the observation that formate is accumulated under conditions of oxygen limitation is interesting, as formate is toxic and typically further metabolised to dihydrogen. However, only few studies in bioreactors approached the analysis of dihydrogen in the context of reactor mixing of *E. coli *cultures [9,10]. Cleland et al. [9] studied the evolution of dihydrogen gas at various oxygen uptake rates in mineral salt medium cultures. The conclusion was that the relative formation of dihydrogen gas was lower than the formation of other anaerobic metabolites including formate. Dihydrogen evolution increased linearly only during the first hour after the oxygen limitation but was then rapidly diminished. The authors proposed that dihydrogen production was occurring with very low rate or that the produced dihydrogen was consumed in another reaction inside the cell.

The disproportionation of formate to carbon dioxide and dihydrogen without nitrate or oxygen as exogenous terminal electron acceptors is catalyzed by the formate hydrogenlyase (FHL) complex, consisting of formate dehydrogenase (FDH, *fdhF *gene product) and of six other proteins encoded by the *hyc *operon. FHL functions as a membrane-integral electron transfer chain which finally releases dihydrogen by the HycE hydrogenase subunit (*hycE*). In *E. coli *exist three FDH isoenzymes, all sharing the same mechanism for formate cleavage. These isoforms are differentially expressed in dependence on the availability of exogenous electron acceptors. Dihydrogen is synthesised by FDH-H only, while the other isoforms, FDH-N and FDH-O, perform the reaction with nitrate as terminal electron acceptor. All three isoenzymes of FDH are molybdo-seleno proteins and HycE is a NiFe hydrogenase. Therefore a functional FHL complex is dependent on trace amounts of molybdenum, selenium, and nickel in the growth medium [11,12].

These essential trace elements for the function of the FHL pathway are generally added into the cultivation medium of anaerobic cultures [13,14]. However, when checking different cultivation media which are used for high cell density cultivation of *E. coli *in small or large bioreactor scales, we experienced that none of them contained selenium or nickel. Only few media contained molybdenum (cf. [3,15-20]).

Oxygen limitation occurs generally in later phases of shake flask cultures and is also experienced by cells in large-scale bioreactors during their passage through the feeding zone. Therefore we considered it highly interesting to study the effects of the addition of the extra trace elements molybdenum, selenium, and nickel on the dynamics of the anaerobic metabolites and the total behaviour of the culture. We expected a lower level of formate accumulation, which would be a positive effect, as formate may be similar toxic to cultures as acetate. However, one also might postulate that the activation of the FHL complex, which leads to release of dihydrogen and carbon dioxide, may negatively affect the carbon yield due to the loss of formate as a re-metabolisable carbon source. Furthermore, the produced carbon dioxide may either positively influence the biomass yield by re-entering the cell's metabolism in carbonylation reactions, or negatively by accumulation in the cultivation medium to toxic concentration.

## Results

In the area of large-scale bioprocessing of *E. coli *the accumulation of formate as a result of imperfect mixing is a widely discussed issue. However, the effect of the additional trace elements which are necessary for a functional formate hydrogen lyase complex, especially selenium and nickel was never considered in this aspect.

**Figure 1:**
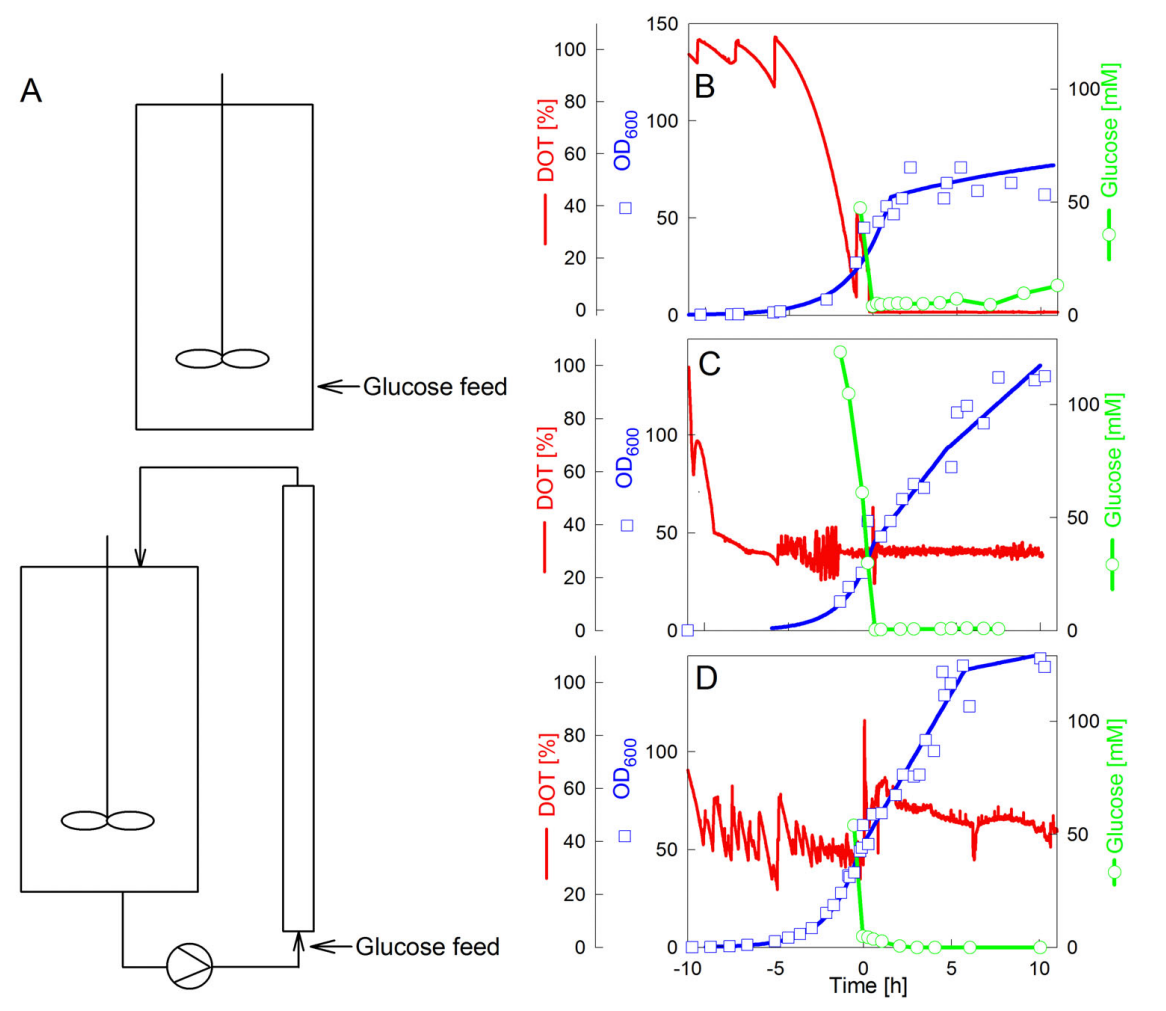
Presentation of different cultivation modes used in this study. (A) Scheme of the two reactor setups used in the study, a standard fed-batch in a stirred tank reactor and a fed-batch performed in a STR-PFR two-compartment reactor system with feeding of the glucose feed solution into the entrance of the PFR. (B-D) illustrate typical cultivation data of *E. coli *W3110 with the different types of cultivation applied in this study. (B) Glucose limited fed-batch cultivation with continuous feed of glucose at a constant feed rate in a single STR (reference). (C) Same as (B) but with a downshift of the DOT by a simultaneous decrease of stirrer speed and aeration rate. (D) Cultivation in a STR-PFR system with feeding of the glucose feed solution into the lower part of the PFR. The graphs show the experimental data for cell density (OD_600_), glucose concentration, and the DOT. All analyses from the STR-PFR system were performed from samples collected by the standard sampling valve in the STR. Zero hours indicates the time of the feed start. The analyses in (C) reflect samples taken from the STR compartment. All cultivations were started with a glucose concentration of 40 g L^-1^.

**Figure 2:**
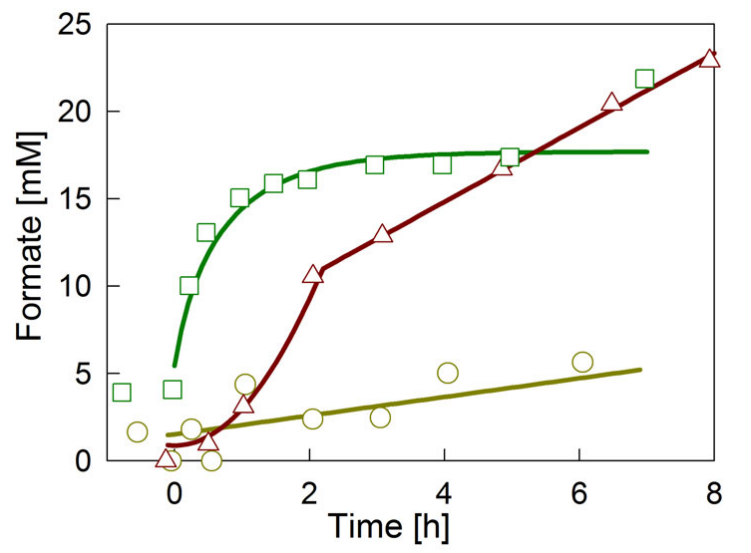
Formate accumulation profiles in the different types of glucose limited fed-batch cultivations of *E. coli *W3110 without supplementation of the trace elements selenium, nickel and molybdenum. The data are from the standard glucose-limited fed-batch reference cultivation (○), from the cultivation with the STR-PFR (△), and from the cultivation with a permanent oxygen down-shift (□). Zero hours indicates the time of the feed start.

The aim of this work was to evaluate the effects of the addition of these trace elements into the cultivation medium. For the studies we selected the commonly used *E. coli *K-12 strain W3110. The control cultivation was a glucose limited fed-batch with constant feeding of a concentrated glucose solution and a DOT above 30% (Figure 1B). Under these conditions the accumulated formate concentration was very low (see Figure 2).

Furthermore, we caused oxygen limitation by different strategies to simulate conditions which are relevant in bioprocesses. Therefore in the first type of cultivation the stirrer and aeration rates were simultaneously set down at the end of the initial batch process, so that the culture run into continuous oxygen limitation (Figure 1C).

For the second type of cultivation a two-compartment scale-down bioreactor was used, consisting of a stirred-tank reactor (STR) and a plug flow reactor (PFR) [5,21,22]. This type of reactor with feeding of the glucose into the lower part of the PFR compartment was shown earlier to be a valuable tool to imitate the feeding zone of a large-scale bioreactor [1,23]. In the set-up which has been used here, about 10% of the cells are steadily exposed to the PFR compartment. As glucose is fed to the entrance of the PFR, the PFR is characterised by a high glucose concentration, i.e. approximately 0.5 to 1.5 g L^-1 ^during the fed-batch phase depending on the glucose feed rate and the pump flow rate through the PFR. Without extra oxygenation the PFR turns at higher cell densities into an anaerobic zone due to the high metabolic activity resulting in the production of typical anaerobic metabolites [3,5]. In the STR compartment, which is kept aerobic and under glucose limitation (Figure 1D), these metabolites are mostly re-consumed and their concentration is low [1,3].

In both scale-down simulators where oxygen limitation occurred either in the whole process permanently or transiently in the PFR compartment, formate was clearly accumulated (Figure 2). In the cultivation with a permanent oxygen downshift formate accumulated relatively fast, but ceased later and formate stayed at a level of about 17 mM. In contrast, formate accumulated continuously during the cultivation in the STR-PFR system in the samples collected from the STR until the end of the cultivation.

The addition of FHL-related trace elements selenium, nickel and molybdenum into the cultivation medium decreased the accumulation of formate in the cultivation medium significantly. This behaviour occurred in both scale-down simulators.

**Figure 3:**
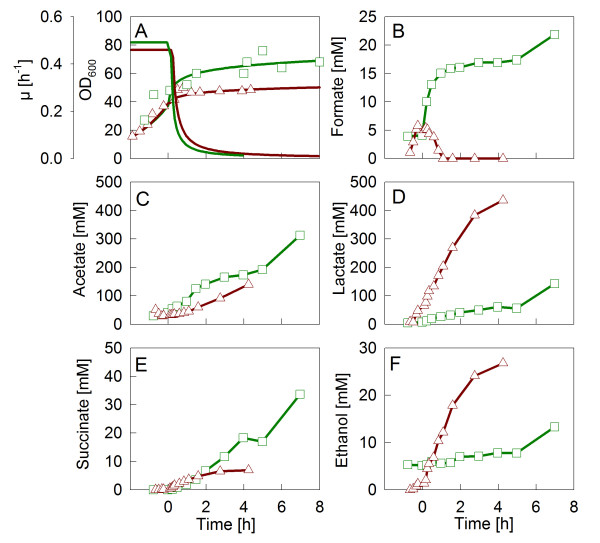
Growth curves and anaerobic metabolite concentrations in fed-batch cultivations of *E. coli *W3110 with a permanent downshift of the DOT at the time of feed start (zero hours). Cultivations were performed with (△ and dashed line) or without (□ and solid line) addition of the trace elements selenium, nickel and molybdenum. (A) Specific growth rate and optical density; (B) accumulation of formate, (C) acetate, (D) lactate, (E) succinate, and (F) ethanol.

In the cultivation with permanent oxygen limitation, when the FHL-related trace elements were added, formate was only slightly accumulated within the first 1.5 hours, but decreased afterwards below the detection limit (Figure 3B). Interestingly, the addition of the FHL-related trace elements affected also the accumulation patterns of the other anaerobic metabolites. Particularly lactate showed a very strong increase. Also changes in the accumulation pattern of the other acids were observed. As shown in Figure 3C, acetate accumulated with a very low rate directly after the oxygen downshift, but approached a constant higher rate about 1 hour after the shift, so that the concentration was only slightly lower compared to the control cultivation at 4 hours after the onset of oxygen limitation. Succinate accumulation was similar with and without the additional trace elements, but its level stayed constant from two hours after the onset of limitation onwards, so that the final level was lower compared to the control cultivation (Figure 3E). The ethanol concentration increased during the whole cultivation (Figure 3F), similar to lactate, but the concentration was only less than one tenth of the lactate concentration. In contrast, ethanol reached a maximum of 10 mM in control cultivations without the addition of the extra trace elements.

It was interesting to observe that the final cell yield of biomass per substrate was lowered by more than one third (see Table 1), from 0.38 g g^-1 ^in the control cultivation to 0.23 g g^-1 ^in the cultivation with addition of the extra trace elements, corresponding to a lower cell dry weight of about 7 g L^-1 ^(Figure 1A). This loss of cell mass is very likely a result of the very high lactate accumulation, which is increased by approximately 36 g L^-1^, but on the other side also may be a result of carbon loss through the FHL-related carbon dioxide production.

**Table 1:** Biomass yield, cell dry weight and side product concentration for *E. coli *W3110 during the different cultivation types on glucose based mineral salt medium.

**Type of cultivation**	**Addition of trace elements**	**Y_X/S_^1 ^(g g^-1^)**		**CDW (g L^-1^)**		**Acetate (mM)**		**Lactate (mM)**		**Formate (mM)**	
		
		**Batch**^2^	**Fed-batch**^3^	**Batch**	**Fed-batch**	**Batch**	**Fed-batch**	**Batch**	**Fed-batch**	**Batch**	**Fed-batch**
Control	+	0.49 (0.01)^4^	n.d.	19.3 (1.1)	n.d.	43.8 (10.1)	n.d.	4.5 (6.3)	n.d.	0.5 (0.7)	n.d.
Control	-	0.48 (0.01)	0.55	18.9 (0.4)	36.4	30.8 (8.4)	29.7	3.8 (3.4)	11.6	1.8 (2.0)	5.0
STR-PFR	+		0.50		37.8		0.1		2.2		0.0
STR-PFR	-		0.45		36.4		4.5		0.0		14.8
Oxygen downshift	+		0.23		20.2		139.7		435.8		0.0
Oxygen downshift	-		0.38		24.7		173.2		60.6		16.9

^1 ^Yield coefficients were calculated for a time period of 4 hours starting with the feeding start. Here Y_X/S _includes also biomass formed from acetate which is consumed during this phase.

^2 ^Batch samples were taken at the end of the exponential growth phase.

^3 ^Fed-batch sample was taken 4 h after the feed start.

^4 ^Numbers in brackets indicate standard deviations between different cultivations.

**Figure 4:**
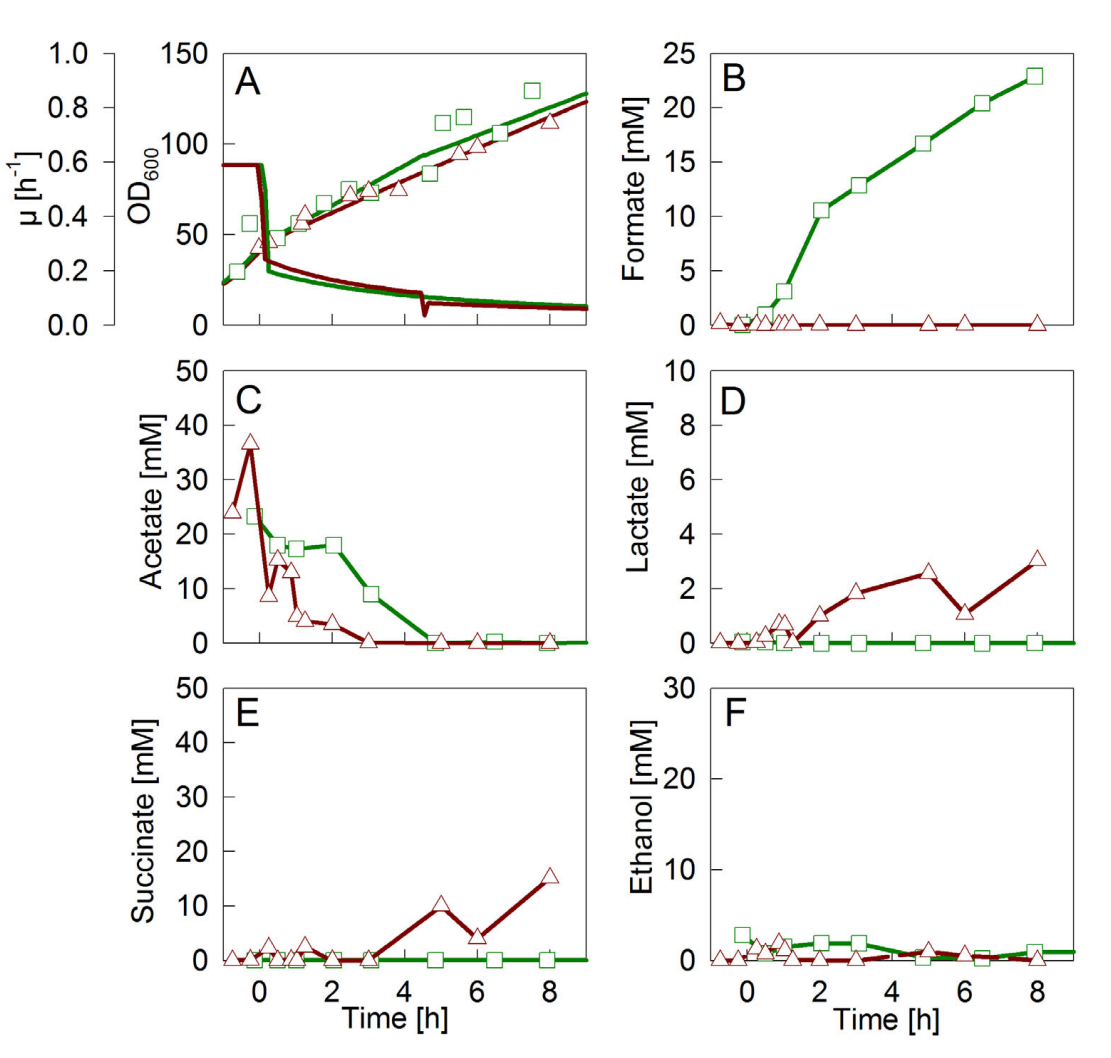
Growth curves and anaerobic metabolite concentrations in fed-batch cultivations of *E. coli *W3110 in the STR-PFR system with (△ and dashed line) or without (□ and solid line) addition of the trace elements selenium, nickel and molybdenum. (A) Specific growth rate and optical density; (B) accumulation of formate, (C) acetate, (D) lactate, (E) succinate, and (F) ethanol. Time of feed start is set to zero hours.

In the STR-PFR two-compartment reactor formate is accumulated in the PFR part of the reactor, where anaerobic conditions occur when no extra oxygen is added. In the STR, under aerobic conditions and glucose limitation, the anaerobic metabolites are consumed. However, formate accumulates during the cultivation as the consumption rate is much lower than the production rate (for earlier results and discussion see [3]). In the STR-PFR system under our cultivation conditions without addition of the extra trace elements, over 20 mM of formate were accumulated in the medium until the end of the cultivation (Figure 4). Approximately the same level of formate was found in a similar scale-down approach by Bylund et al. [24]. In our case, addition of the extra trace elements resulted in a significant decrease; formate stayed very low during the whole fermentation (see Figure 4B). In the cultivation with addition of the extra trace elements, the maximum concentration of formate detected from the STR compartment was only 0.2 mM in the end of the batch phase and remained below that until the end of the cultivation. The effect on the other metabolites in this case was minor (Figure 4C–F). All of them only reached very low concentrations, one to three orders of magnitude lower than in the cultivation with the oxygen downshift, due to their relatively high consumption rates in the aerobic STR compartment (see discussion in [1,3]). As expected, the biomass yield per glucose was lower in the cultivations with the STR-PFR two-compartment reactor system compared to the control cultivation. However, interestingly the biomass yield was somewhat higher in the STR-PFR cultivation with addition of the extra trace elements (0.50 g g^-1 ^with addition of the extra trace elements, 0.45 g g^-1 ^without addition of these trace elements, cf. Table 1).

## Discussion

Commonly used mineral salt media for bioreactor cultivations of the bacterium *E. coli *are designed for aerobic growth, i.e. they do not contain some of the chemical elements which are necessary for the anaerobic growth of *E. coli*. Selenium, nickel, and molybdenum are cofactors of the formate hydrogen lyase complex, an enzyme system which degrades formate to carbon dioxide and hydrogen. Hydrogen is either released as dihydrogen or transferred to nitrate as terminal electron acceptor, which however is not commonly contained in fed-batch cultivation media.

Formate accumulation has been an interesting observation and discussion issue in large-scale fed-batch cultivations and in recombinant cultures. Furthermore, also shake flask cultivations can easily run into oxygen limitation. Therefore we wanted to investigate whether the current situation of not including these trace elements in cultivation media provides a clear benefit, or whether these trace elements are simply not used due to historical reasons.

As formate should have a similar toxic effect in cultures as described for acetate, the suppression of formate accumulation might be an important issue, despite the benefit of having formate as an easily detectable indicator for anaerobic zones in a bioreactor. Surprisingly, despite the generally good knowledge on the anaerobic metabolism of *E. coli*, we could not find any answer to these questions in the literature and therefore we decided to perform some key experiments to investigate the pros and cons for the use of these additional trace elements.

For this study we used two different simulators which imitate different scenarios of oxygen limitation which typically occur in *E. coli *cultures. Firstly, we performed a downshift of the stirrer rate during a fed-batch cultivation to provoke permanent oxygen limitation after the shift. As the shift was performed under glucose excess, this procedure mimicked the conditions in a shake flask. Secondly, we used an STR-PFR two-compartment reactor which imitates a large-scale bioreactor with limited mixing in the feed zone [5,23,25]. Both simulators were performed either with or without addition of the trace elements selenium, nickel and molybdenum.

In the control experiments without addition of the extra trace elements the cultures behaved as expected. In the simulator with permanent oxygen limitation, the biomass yield on glucose decreased significantly and acetate accumulated as the major organic acid in the cultivation medium, followed by lactate. The other anaerobic metabolites, succinate, formate, and ethanol accumulated to significantly lower concentrations. In contrast, oscillatory oxygen limitation in the STR-PFR system resulted in the accumulation of formate only, acetate which accumulated during the batch phase, before connection of the plug flow reactor, decreased continuously until the end of the cultivation. Also cultivation in this system had not a major influence on the biomass yield, the final cell density was only slightly, but significantly lower compared to a cultivation which was performed only in a stirred tank reactor.

The addition of selenium, nickel, and molybdenum drastically decreased the accumulation of formate in both simulators, which clearly indicates, that the observation that formate accumulates under certain conditions in *E. coli *fed-batch cultures, is simply due to the impaired function of FHL enzyme complex, when the necessary cofactors are not available.

An interesting and unexpected result was the very high accumulation of lactate during permanent oxygen limitation to about 40 g L^-1 ^which was only seen when the extra trace elements were added. This observation provokes the question by which metabolic pathway this lactate is synthesised. Aside from the reaction of lactate dehydrogenase yielding D(-) lactate from pyruvate, alternatively the accumulated lactate could origin from the methylglyoxyl pathway starting with dihydroxyacetone phosphate (DHAP) and result in a mixture of L(+) and D(-) lactate. The latter route is an energetically less favourable alternative to the second phosphorylation step in the lower part of the glycolysis which has been described to be active in *E. coli *and *Klebsiella aerogenes *under anaerobic growth [26-28] and during aerobic growth when metabolic imbalances occurred, e.g. induced by a glucose pulse or by deregulation of the uptake of different carbon compounds by mutation and/or by addition of cyclic AMP (reviewed by [29]). Interestingly, in our study the accumulating lactate is exclusively D-Lactate as has been verified by L-lactate analysis with an L-lactate biosensor (YSI analyser, Yellow Springs Instruments). The L-lactate concentration was below 100 mg L^-1 ^(data not shown), which suggests that the lactate is directly synthesised from pyruvate by D-lactate-dehydrogenase.

In connection to the high accumulation of lactate in the experimental set-up with permanent oxygen limitation, the cell yield was significantly decreased. This would be an important issue if one would consider the general application of the extra trace elements in *E. coli *cultures. The fear of high lactate accumulation and lower biomass yield as a consequence of oxygen limitation would be definitely a problematic issue.

Interestingly, in contrast to the cultures with a permanent oxygen limitation, in the oscillating simulator, the STR-PFR two-compartment reactor, no negative impact of the addition of the extra trace elements was observed, but the yield coefficient for biomass production on glucose was even slightly higher when the extra trace elements were added (cf. Figure 1). This might suggest that some of the produced carbon dioxide is again re-metabolised in carboxylation reactions. Furthermore, in case of the STR-PFR system with addition of the extra trace elements there was no special accumulation of lactate or any of the other fermentation side products detected in the cultivation medium, which indicates a well functioning lower part of the glycolysis. The experiments in the two-compartment scale-down simulator indicate that the addition of the extra trace elements could be worth trying to decrease the concentration of short chain organic acids, such as formate and acetate, if there are no other simple ways to improve the mixing.

## Conclusion

The accumulation of formate in partially oxygen limited cultivations can be prevented by adding the trace elements selenium, nickel, and molybdenum, necessary for the function of FHL complex, into the cultivation medium. With these trace elements formate decreased close to the detection limit in the two-compartment scale-down simulator. In comparison, more than 20 mM of formate accumulated during cultivations in the two-compartment system without these trace elements. As this two-compartment reactor has been earlier shown to provide a good estimation of the effects of limited mixing in large-scale bioreactors [1,23], our results may clearly suggest beneficial effects of the addition of selenium, nickel, and molybdenum in large-scale bioprocesses, where the cells are exposed to oscillations in the glucose and oxygen levels. We could also not observe any other negative effects on any bioprocess parameters when the cultivation was either performed as well mixed oxygen-limited fed-batch or when it was exposed to glucose and oxygen oscillations in the scale-down bioreactor (cf. Table 1).

However in contrast, media which contain these trace elements may be problematic under cultivation conditions with permanent oxygen limitation, which may be relevant for small-scale shaking cultures. Under permanent oxygen limitation the positive effect of the reduction of formate was outcompeted by an extremely high accumulation of lactate and a severe drop of the biomass yield.

## Methods

### Strains

The strain used in this study was *Escherichia coli *K-12 strain W3110 [F^- ^λ^-^IN (*rrnD-rrnE*)1]. The stock solution of the strains was stored in 15% [v/v] glycerol solution at -70°C.

### Precultures

The first preculture was performed in 20 mL of LB medium in 100 mL Erlenmeyer flasks for 8 to 16 hours. The second preculture was performed in 1000 mL baffled shake flasks containing 200 mL of mineral salt medium with an initial glucose concentration of 10 g L^-1^. Both precultures were cultivated at 37°C on a rotary shaker with a rate of 160 rpm.

### Bioreactor fed-batch cultures

The cultivations were performed in a Biostat C 15 L bioreactor with the DCU-3 controlling unit and MFCS-win supervisory system (Sartorius) with an initial working volume of 8 L or 10 L in oxygen downshift and two-compartment scale-down cultivations, respectively. The stirred tank reactor was equipped with 3 equally spaced standard six-blade Rushton turbines and four baffles. The cultures were inoculated to an initial OD_600 _of 0.05 to 0.1. The cultivation temperature was always 37°C and the pH was kept at 7.0 by controlled feeding of 25% ammonium hydroxide.

The cultivation medium was a defined mineral salt medium with an initial glucose concentration of 40 g L^-1^. The mineral salt medium was prepared in distilled water containing in g L^-1^: K_2_HPO_4 _14.6, NaH_2_PO_4 _× 2 H_2_O 3.6, Na_2_SO_4 _2.0, (NH_4_)_2_SO_4 _2.47, NH_4_Cl 0.5, (NH_4_)_2_-H-citrate 1.0, and 0.1 mL antifoam 204 (Sigma). After heat sterilization 2 mL L^-1^of 1 M MgSO_4 _and 2 mL L^-1 ^trace element solution [30] were added as well as 0.1 g L^-1 ^of thiamine hydrochloride through a sterile filter (0.2 μm). To investigate the effects of addition of selenium, nickel and molybdenum, these extra trace elements were added whenever indicated in the following concentrations: 0.17 mg L^-1 ^Na_2_SeO_3 _× 5 H_2_O; 0.24 mg L^-1 ^Na_2_MoO_4_, and 1.45 mg L^-1^Ni(NO_3_)_2 _× 6 H_2_O. These elements were only added at the beginning of the cultivation. The feed solution contained 650 g L^-1 ^glucose, but no extra salts. During all fed-batch cultivations extra 2 ml L^-1 ^of a 1 M MgSO_4 _solution was added during the cultivation approximately for every 10 units increase in OD_600_.

The reference cultivation was performed as glucose-limited fed-batch with the DOT controlled above 30%. The feeding rate was 76 g of glucose per hour.

### Scale-down simulators

Cultivations with permanent oxygen limitation after a DOT downshift experiments were performed in the same way as the control glucose-limited fed-batch culture with an initial liquid volume of 8 L. Oxygen limitation was caused by a sudden decrease of stirrer rate from 1100 (± 100) rpm down to 500 rpm. The aeration rate was kept at 0.85 (± 0.1) vvm. Glucose feeding was initiated shortly after the DOT downshift, and in difference to the control cultivation the feed rate was manually corrected to keep glucose concentration above 0.5 g L^-1 ^for avoiding glucose limitation.

The two-compartment reactor consisted of a regular stirred tank reactor (STR) linked in series to a plug flow reactor (PFR). The PFR was made up of 4 equally sized glass cylinders each containing a set of removable stainless steel static mixer modules (Kenics, Chemineer, Derby, UK) which gave a total volume of about 1 L. The PFR has been described and characterized in detail earlier [25]. The only difference in our set up was that the PFR cylinders were placed as 2 × 2 cylinders which are interconnected by a polymer tubing so that the liquid was pumped from the upper part of the first cylinder to the low entrance of the second one.

The feed solution was pumped to the entrance of the PFR, below the first glass tube in a 90° angle to provide fast mixing of the concentrated glucose feed into the circulating broth. The broth was pumped from the STR into the PFR loop using a peristaltic pump (Millipore) with the rate 1 L min^-1^. As the volume of the plug flow compartment was approximately 1 L, the average residence time for one circle in the loop was approximately 60 sec. The PFR circulation was started at an OD_600 _of about 35, shortly before the batch glucose was exhausted. The glucose feed solution was fed with a rate of 110 g L^-1 ^into the lower entrance of the PFR compartment with the aim of having glucose excess in PFR and limitation in STR compartment. In both cultivations the glucose concentration in the STR compartment stayed below 0.1 g L^-1 ^after the feed start and between 1 and 2 g L^-1 ^in the PFR compartment. The DOT in the STR compartment was kept above 30%.

All analyses from the STR-PFR system were preformed from samples collected by the standard sampling valve in the STR and the on-line DOT values are from the oxygen electrode which was placed in the STR.

### Analysis of cell growth

Cell growth was monitored spectrophotometrically by measurement of the optical density at 600 nm (OD_600_) and cell dry weight. The specific growth rate μ was calculated from the fitted OD_600 _values. One unit of OD_600 _corresponds to a cell dry weight of 0.3 g L-1.

### Analysis of organic acids and glucose

Formate and other anaerobic metabolites were analyzed from the medium samples. Cultivation broth (1 mL) was centrifuged at 16,000 × g for 3 min at +4°C. The supernatant was immediately frozen in liquid nitrogen. Samples were stored at -20°C until analysis. Samples were thawn on ice and incubated at 80°C for 5 min to solubilise possible precipitates and finally filtered with 0.2 μm filters before injection to HPLC.

The HPLC analysis was performed with a Merck-Hitachi HPLC-system (Model D-7000) and ICSep COREGEL 87H3 organic acids column (Transgenomic Inc., Omaha, U.S.A.). 0.01 N H_2_SO_4 _was used as running buffer. Organic acids were quantified with a UV-VIS detector (L-4250, Merck Hitachi) at 210 nm, glucose and ethanol with a differential refractometer (RI-71, Merck) at 190 nm. The analysis software was D-7000 HPLC System Manager (version 3.1.1, Hitachi).

During the cultivation glucose was monitored with a YSI 2700 analyser (Yellow Springs Instruments) from samples centrifuged (16,000 × g, 4°C, 3 min) and filtered (0.2 μm) samples.

## Competing interests

The authors declare that they have no competing interests.

## Authors' contributions

JS carried out the experiments and drafted the manuscript. KU participated in the experiments with the two-compartment reactor. PN conceived of the study, and participated in its design, coordination, and drafting of the manuscript. All authors read and approved the final manuscript.

## References

[B1] Enfors SO, Jahic M, Rozkov A, Xu B, Hecker M, Jürgen B, Krüger E, Schweder T, Hamer G, O'Beirne D, Noisommit-Rizzi N, Reuss M, Boone L, Hewitt C, McFarlane C, Nienow A, Kovacs T, Trägardh C, Fuchs L, Revstedt J, Friberg PC, Hjertager B, Blomsten G, Skogman H, Hjort S, Hoeks F, Lin HY, Neubauer P, van der LR, Luyben K, Vrabel P, Manelius (2001). Physiological responses to mixing in large scale bioreactors. J Biotechnol.

[B2] Vasala A, Panula J, Bollok M, Illmann L, Hälsig C, Neubauer P (2006). A new wireless system for decentralised measurement of physiological parameters from shake flasks. Microb Cell Fact.

[B3] Xu B, Jahic M, Blomsten G, Enfors SO (1999). Glucose overflow metabolism and mixed-acid fermentation in aerobic large-scale fed-batch processes with *E. coli*. Appl Microbiol Biotechnol.

[B4] LONGMUIR IS (1954). Respiration rate of bacteria as a function of oxygen concentration. Biochem J.

[B5] Neubauer P, Häggstrom L, Enfors SO (1995). Influence of substrate oscillations on acetate formation and growth yield in *E. coli* glucose-limited fed-batch cultivations. Biotechnol Bioeng.

[B6] Bylund F, Castan A, Mikkola R, Veide A, Larsson G (2000). Influence of scale-up on the quality of recombinant human growth hormone. Biotechnol Bioeng.

[B7] Lara AR, Vazquez-Limon C, Gosset G, Bolivar F, Lopez-Munguia A, Ramirez OT (2006). Engineering *E. coli* to improve culture performance and reduce formation of by-products during recombinant protein production under transient intermittent anaerobic conditions. Biotechnol Bioeng.

[B8] Castan A, Enfors SO (2002). Formate accumulation due to DNA release in aerobic cultivations of *E. coli*. Biotechnol Bioeng.

[B9] Cleland N, Larsson G, Enfors SO (1990). Characterization of A Biological Test System for Studies on Insufficient Mixing in Bioreactors - H2 evolution from *E. coli*. Bioprocess Engineering.

[B10] Larsson G, Enfors SO (1993). Kinetics of *Escherichia coli* hydrogen production during short-term repeated aerobic-anaerobic fluctuations. Bioprocess Engineering.

[B11] PINSENT J (1954). The need for selenite and molybdate in the formation of formic dehydrogenase by members of the coli-aerogenes group of bacteria. Biochem J.

[B12] Sawers RG (2005). Formate and its role in hydrogen production in *E. coli*. Biochem Soc Trans.

[B13] Hörnsten EG (1995). On culturing *E. coli* on a mineral salts medium during anaerobic conditions. Bioprocess Engineering.

[B14] Yoshida A, Nishimura T, Kawaguchi H, Inui M, Yukawa H (2005). Enhanced hydrogen production from formic acid by formate hydrogen lyase-overexpressing *E. coli* strains. Appl Environ Microbiol.

[B15] Hellmuth K, Korz DJ, Sanders EA, Deckwer WD (1994). Effect of growth rate on stability and gene expression of recombinant plasmids during continuous and high cell density cultivation of *E. coli* TG1. J Biotechnol.

[B16] Riesenberg D, Menzel K, Schulz V, Schumann K, Veith G, Zuber G, Knorre WA (1990). High cell density fermentation of recombinant *E. coli* expressing human interferon alpha 1. Appl Microbiol Biotechnol.

[B17] Bauer S, Shiloach J (1974). Maximal exponential growth rate and yield of *E. coli *obtainable in a bench-scale fermentor. Biotechnol Bioeng.

[B18] Yoon SK, Kang WK, Park TH (1994). Fed-batch operation of recombinant *E. coli* containing trp promoter with controlled specific growth rate. Biotechnol Bioeng.

[B19] Fieschko J, Ritch T (1986). Production of human alpha-consensus interferon in recombinant *E. coli*. Chemical Engineering Communications.

[B20] Mori H, Yano T, Kobayashi T, Shimizu S (1979). High density cultivation of biomass in fed-batch system with DO-stat. J Chem Engin Japan.

[B21] George S, Larsson G, Olsson K, Enfors SO (1998). Comparison of the Baker's yeast process performance in laboratory and production scale. Bioprocess Engineering.

[B22] Neubauer P, Åhman M, Törnkvist M, Larsson G, Enfors SO (1995). Response of guanosine tetraphosphate to glucose fluctuations in fed-batch cultivations of *E. coli*. J Biotechnol.

[B23] Hewitt CJ, Nienow AW (2007). The scale-up of microbial batch and fed-batch fermentation processes. Adv Appl Microbiol.

[B24] Bylund F, Collet E, Enfors SO, Larsson G (1998). Substrate gradient formation in the large-scale bioreactor lowers cell yield and increases by-product formation. Bioprocess Engineering.

[B25] George S, Larsson G, Enfors SO (1993). A scale-down 2-compartment reactor with controlled substrate oscillations - metabolic response of *Saccharomyces cerevisiae*. Bioprocess Engineering.

[B26] Grabar TB, Zhou S, Shanmugam KT, Yomano LP, Ingram LO (2006). Methylglyoxal bypass identified as source of chiral contamination in L(+) and D(-)-lactate fermentations by recombinant *E. coli*. Biotechnol Lett.

[B27] Zhu MM, Skraly FA, Cameron DC (2001). Accumulation of methylglyoxal in anaerobically grown *E. coli* and its detoxification by expression of the Pseudomonas putida glyoxalase I gene. Metab Eng.

[B28] Simons JA, Snoep JL, Feitz S, Teixeira de Mattos MJ, Neijssel OM (1992). Anaerobic 2-ketogluconate metabolism of Klebsiella pneumoniae NCTC 418 grown in chemostat culture: involvement of the pentose phosphate pathway. J Gen Microbiol.

[B29] Weber J, Kayser A, Rinas U (2005). Metabolic flux analysis of *E. coli* in glucose-limited continuous culture. II. Dynamic response to famine and feast, activation of the methylglyoxal pathway and oscillatory behaviour. Microbiology.

[B30] Holme T, Arvidson S, Lindholm B, Pavlu B (1970). Enzymes: laboratory-scale production. Process Biochem.

